# A rare association of caecal volvulus and intestinal malrotation causing an acute abdomen: Case report

**DOI:** 10.1016/j.amsu.2021.102357

**Published:** 2021-04-30

**Authors:** Youssef Chaker, Yacine Ouadi, Ahmed Ben Mahmoud, Anis Haddad, Houcine Magherbi, Montasser Kacem

**Affiliations:** a Department of Surgery A La Rabta, Tunis, Tunisia; b Faculty of Medicine of Tunis, Tunis El Manar University, Tunis, Tunisia

**Keywords:** Case report, Caecal volvulus, Intestinal malrotation, Acute abdomen, General surgery

## Abstract

**Introduction:**

and importance: Caecal volvulus represents 30% of colonic volvulus. It happens due to torsion or hyperflexion of a hypermobile caecum. Usually it is secondary to an axial rotation of the caecum and the ileum around the mesentery. On the other hand Intestinal malrotation occurs due to incomplete or faulty rotation and fixation of the gut during fetal life. The occurrence of these two anomalies together is scarse which makes this case report interesting.

**Case presentation:**

A 75 year old man with medical history of terminal kidney failure, presented to the emergency room with an intestinal obstruction syndrome. On examination the patient had a distended abdomen with tenderness in the left upper quadrant. Biology found an important biological inflammatory syndrome with hyperleukocytosis and elevated CRP. Plain X-ray of the abdomen in erect posture showed an air fluid colonic level in the left hypochondrium. CT scan showed signs of caecal volvulus with intestinal malrotation. A brief reanimation and nasogastric aspiration couldn't solve the problem therefore emergency laparotomy was needed ileocaecal resection was performed associated with LADD's procedure in order to treat both anomalies and prevent further gut volvulus.

**Clinical discussion:**

Despite it's rareness, caecul volvulus represents the second cause of large bowel volvulus just behind sigmoid volvulus. Intestinal malrotation in adults subjects is estimated to occur in 0.2–0.5%.The uniqueness of our case is that these two anomalies were associated in such a way that it made both the diagnosis and the therapy even more difficult. Abdominal CT has become mandatory for pre-operative diagnosis of intestinal volvulus. Surgery is the gold standard treatment for caecal volvulus. The usual options are manual detorsion, carcopexy, caecostomy and colectomy.

**Conclusion:**

This case reports a rare association of a caecum volvulus with intestinal malrotation that emphasis the place of modern technologies such as CT scan in order to achieve correct preoperative diagnosis. We also describe our approach to this uncommon surgical emergency in order to provide an efficient treatement.

## Highlights

• Caecal volvulus associated with intestinal malrotation is rare.
• CT scan is mandatory for preoperative diagnosis of caecal volvulus.
• Surgery is the only permanent solution.
• Association of procedures for the caecal volvulus and the intestinal malrotation is the only prevention from recurrence.

## 1 Introduction

Caecal volvulus represents 30% of colonic volvulus. It happens due to torsion or hyperflexion of a hypermobile caecum. Usually it is secondary to an axial rotation of the caecum and the ileum around the mesentery [2]. On the other hand Intestinal malrotation occurs due to incomplete or faulty rotation and fixation of the gut during fetal life [3]. The occurrence of these two anomalies together is scarse which makes this case report interesting.

This case report has been reported in line with the SCARE Criteria [1].

## 2 Case report

A 75 year old man with medical history of terminal kidney failure, who presented to the emergency department with abdominal pain and bloating associated with bilious vomiting evolving for 3 days. He reported recurrent abdominal pain and bloating since childhood kept without investigation.

**Fig. 1:**
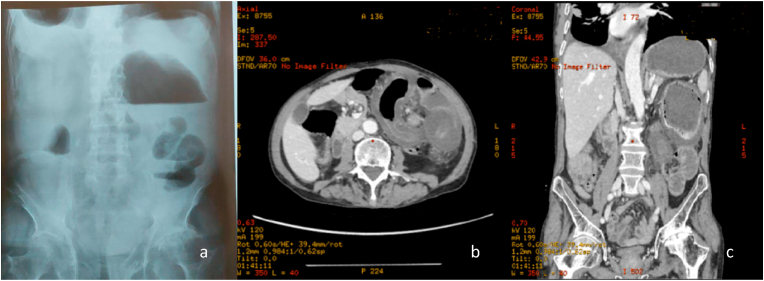
(a) Plain X-ray of the abdomen in erect posture with an air fluid colonic level in the left hypochondrium (b) CT SCAN showing the whirl sign (c) CT scan showing the dilated caecum in the left hypochondrium.

Upon examination, the patient was calm, afebrile, with normal vital signs. The left abdomen was distended with tenderness in the left upper quadrant. Laboratory tests showed a high c-reactive protein level (150mg/l) and a high white blood cell count (20000/μl). No anemia nor hemostasis disorders were noticed. Plain X-ray of the abdomen in erect posture showed an air fluid colonic level in the left hypochondrium (Fig. 1 a).

Emergency Ct-Scan of the abdomen showed a dilated caecum located in the left hypochondrium measuring up to a diameter of 12 cm. Whirl sign and transposition of superior mesenteric artery to the right of superior mesenteric vein on CT scan made the diagnostic clear of caecal volvulus on intestinal malrotation. A bird beak sign was also found.

The patient was admitted in a surgical reanimation unit, nasogastric aspiration brought 400 ml of fecaloid liquid.

**Fig. 2:**
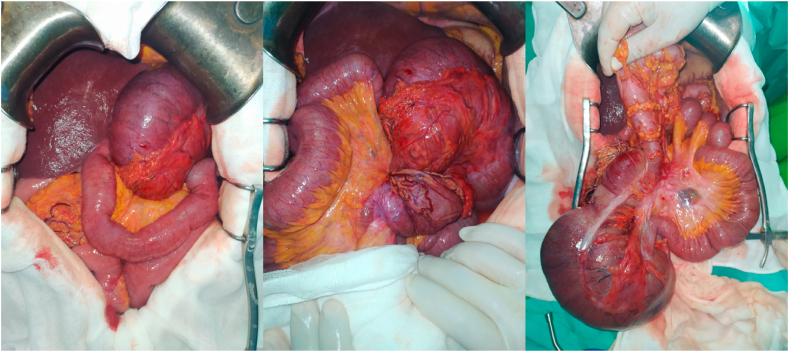
(a) in operative view with a dilated caecum in the left hypochondrium (b) twist of the volvulus around the ileocolic artery (c) in operative view after devolvulation.

**Fig. 3:**
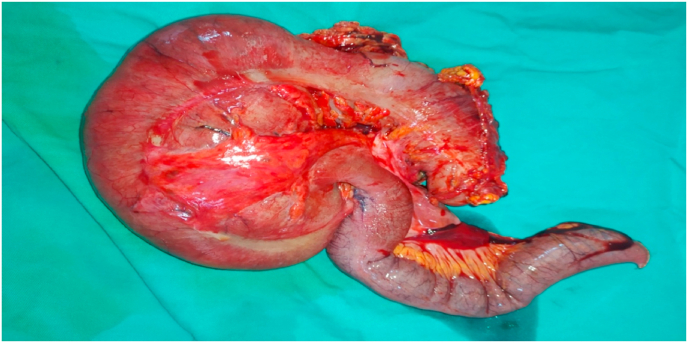
ileocaecal resection.

We decided to perform an emergency surgery. Surgery was performed by an attending physician with 12 years surgical experience in our teaching hospital. The caecum was in the left hypochondrium hugely distended and contained signs of prepeforation such as deperitonisation lesions (Fig. 2 a). Caecal volvulus was seen (Fig. 2 b). Clockwise de-rotation of volvulus was performed. Upon devolvulation All the small bowels were located in the left side of the abdomen and all the colonic segments were in the right side (Fig. 2 c). We deducted that the patient had two anomalies: the first being an intestinal malrotation that made the caecum located originally in the upper right quadrant of the abdomen and the second was a poor peritoneum fixation of the caecum that made the volvulus possible due to its high mobility. We decided upon these findings to perform an ileocaecal resection (Fig. 3) with immediate ileocolic anastomosis using GEA 60 charges followed by LADD's procedure. Antibiotics prophylaxis was given for 48 hours post operatively using cefazolin intravenously at the dosage of 1gr three times a day. Venous thromboembolism prophylaxis was given using enoxaparin subcutaneously at the dosage of 4000 UI once a day.

The postoperative period was uneventful, and the patient was discharged on Day 6. A Follow up examination was done 2 weeks and then 30 days after surgery and was without abnormalities. There was no need for further biological analysis or imaging.

## 3 Discussion

Despite it's rareness, caecul volvulus represents the second cause of large bowel volvulus just behing sigmoid volvulus. It accounts for almost 30% of colonic volvulus (2). It is due to the rotation or flexion of a poorly fixed caecum due to a default in colonic mesentary fusion during the fetal period [3]. Intestinal malrotation is due to a default in rotation and fixation of the small bowels during the first trimester of fetal life. Intestinal malrotation in adults subjects is estimated to occur in 0.2–0.5% [4,5].

The uniqueness of our case is that these two anomalies were associated in such a way that it made both the diagnosis and the therapy even more difficult.

Since both caecal volvulus and intestinal malrotation are scarce, clinical diagnosis is challenging. Therefore abdominal CT has become mandatory for pre-operative diagnosis of intestinal volvulus. The ‘whirl’ sign, beak sign, intestinal twisting, and the direction of the volvulus have been described in literature [6].

Surgery is the gold standard treatment for caecal volvulus. The usual options are manual detorsion, caecopexy, caecostomy and colectomy. Choosing from one of those options will depend on operative findings [2]. The best long-term results have been with resection and primary anastomosis. Although we did find in litterature some cases of laparoscopic caecopexy that showed promising results [7].

Endoscopic devolvulation is possible but this procedure fails in over 70% of the cases [8].

In case of association with intestinal malrotation we do recommend treating both anomalies by performing a LADD's procedure to prevent the venue of midgut volvulus in the future.

## 4 Conclusion

This case reports a rare association of a caecum volvulus with intestinal malrotation that emphasis the place of modern technologies such as CT scan in order to achieve correct preoperative diagnosis. We also describe our approach to this uncommon surgical emergency in order to provide an efficient treatement.

In this rare case of caecal volvulus associated with malrotation, detorsion, ileocaecal resection with primary anastomosis and repositioning treated the condition.

## Ethical approval

There is no ethical committee in our country (Not applicable for this manuscript).

## Sources of funding

No sources of funding.

## Author contribution

All authors contributed to the study design.

CHAKER.Y (The operator), performed the surgery. YO, ABM wrote the manuscript draft. MK, HM and AH read and corrected the manuscript. All authors read and approved the final manuscript.

## Registration of research studies

It is a case report.

## Guarantor

OY.

## Consent for publication

Written informed consent was obtained from the patient for publication of this case report and any accompanying images. A copy of the written consent is available for review by the Editor-in-Chief of this journal on request.

## Provenance and peer review

Not commissioned, externally peer reviewed.

## Declaration of competing interest

All authors declare that they have no any conflicts of interest.

## References

[1] Agha R.A., Franchi T., Sohrabi C., Mathew G., Kerwan A., SCARE Group (déc 2020). The SCARE 2020 guideline: updating consensus surgical CAse REport (SCARE) guidelines. Int. J. Surg. Lond. Engl..

[2] Habre J., Sautot-Vial N., Marcotte C., Benchimol D. (nov 2008). Caecal volvulus. Am. J. Surg..

[3] Samuel M., Boddy S.A., Nicholls E., Capps S. (avr 2000). Large bowel volvulus in childhood. Aust. N. Z. J. Surg..

[4] Frantzides C.T., Cziperle D.J., Soergel K., Stewart E. (févr 1996). Laparoscopic ladd procedure and cecopexy in the treatment of malrotation beyond the neonatal period. Surg. Laparosc. Endosc..

[5] Mazziotti M.V., Strasberg S.M., Langer J.C. (août 1997). Intestinal rotation abnormalities without volvulus: the role of laparoscopy. J. Am. Coll. Surg..

[6] Delabrousse E., Sarliève P., Sailley N., Aubry S., Kastler B.A. (nov 2007). Cecal volvulus: CT findings and correlation with pathophysiology. Emerg Radiol..

[7] Madiba T.E., Thomson S.R. (févr 2002). The management of cecal volvulus. Dis. Colon Rectum.

[8] Theuer C., Cheadle W.G. (mars 1991). Volvulus of the colon. Am Surg..

